# Risk Factors for Carbapenem-Resistant *Enterobacterales* Clinical Treatment Failure

**DOI:** 10.1128/spectrum.02647-22

**Published:** 2023-01-09

**Authors:** Nicholas Rebold, Abdalhamid M. Lagnf, Sara Alosaimy, Dana J. Holger, Paige Witucki, Andrew Mannino, Michelle Dierker, Kristen Lucas, Ashlan J. Kunz Coyne, Amer El Ghali, Kaylee E. Caniff, Michael P. Veve, Michael J. Rybak

**Affiliations:** a College of Pharmacy and Health Sciences, Wayne State University, Detroit, Michigan, USA; b Anti-Infective Research Laboratory, Department of Pharmacy Practice, College of Pharmacy and Health Sciences, Wayne State University, Detroit, Michigan, USA; c Department of Pharmacy, Henry Ford Hospital, Henry Ford Health System, Detroit, Michigan, USA; d Detroit Receiving Hospital, Detroit Medical Center, Detroit, Michigan, USA; e School of Medicine, Wayne State University, Detroit, Michigan, USA; Emory University School of Medicine

**Keywords:** carbapenem resistance, *Enterobacterales*, risk factors, clinical failure, ceftazidime-avibactam, meropenem-vaborbactam, CRE, *Enterobacteriaceae*, dialysis, *Klebsiella*, beta-lactams, carbapenems, clinical therapeutics

## Abstract

The Centers for Disease Control and Prevention (CDC) categorized carbapenem-resistant *Enterobacterales* (CRE) infections as an “urgent” health care threat requiring public attention and research. Certain patients with CRE infections may be at higher risk for poor clinical outcomes than others. Evidence on risk or protective factors for CRE infections are warranted in order to determine the most at-risk populations, especially with newer beta-lactam/beta-lactamase inhibitor (BL/BLI) antibiotics available to treat CRE. We aimed to identify specific variables involved in CRE treatment that are associated with clinical failure (either 30-day mortality, 30-day microbiologic recurrence, or clinical worsening/failure to improve throughout antibiotic treatment). We conducted a retrospective, observational cohort study of hospitalized patients with CRE infection sampled from 2010 to 2020 at two medical systems in Detroit, Michigan. Patients were included if they were ≥18 years old and culture positive for an organism in the *Enterobacterales* order causing clinical infection with *in vitro* resistance by Clinical and Laboratory Standards Institute (CLSI) breakpoints to at least one carbapenem. Overall, there were 140 confirmed CRE infections of which 39% had clinical failure. The most common infection sources were respiratory (38%), urinary (20%), intra-abdominal (16%), and primary bacteremia (14%). A multivariable logistic regression model was developed to identify statistically significant associated predictors with clinical failure, and they included Sequential Organ Failure Assessment (SOFA) score (adjusted odds ratio [aOR], 1.18; 95% confidence interval [CI], 1.06 to 1.32), chronic dialysis (aOR, 5.86; 95% CI, 1.51-22.7), and Klebsiella pneumoniae in index culture (aOR, 3.09; 95% CI, 1.28 to 7.47). Further research on CRE infections is needed to identify best practices to promote treatment success.

**IMPORTANCE** This work compares carbapenem-resistant *Enterobacterales* (CRE) infections using patient, clinical, and treatment variables to understand which characteristics are associated with the highest risk of clinical failure. Knowing which risk factors are associated with CRE infection failure can provide clinicians better prognostic and targeted interventions. Research can also further investigate why certain risk factors cause more clinical failure and can help develop treatment strategies to mitigate associated risk factors.

## INTRODUCTION

Antimicrobial resistance is considered to be one of the greatest threats to global health, with antimicrobial-resistant pathogens, such as carbapenem-resistant Enterobacterales (CRE) in the United States costing over an estimated $130 million annually in attributable costs before discharge (1, 2). The Centers for Disease Control and Prevention (CDC) assigned CRE the highest threat level declaring urgent public attention, and the rising prevalence of carbapenem resistance has increased over time from 6% in 2000 to 11% in 2017 (3, 4). Previously, nephrotoxic antibiotics, such as polymyxins and aminoglycosides, had been commonly utilized in treatment regimens to combat these resistant infections (5, 6). However, newer beta-lactam/beta-lactamase inhibitor (BL/BLI) antibiotics developed to overcome resistance mechanisms in carbapenemase-producing CRE, such as ceftazidime-avibactam (CZA) and meropenem-vaborbactam (MVB), have shown improved safety with noninferior efficacy outcomes compared with older agents (7–13).

Studies on specific risk factors associated with clinical failure in CRE treated with newer BL/BLIs are sparse. Previous studies have suggested that renal replacement therapy, respiratory tract infections, prolonged time to active therapy, immunocompromised status, higher severity measured through scores (acute physiology and chronic health evaluation II score [APACHE II], Sequential Organ Failure Assessment [SOFA], and INCREMENT-CPE score [INCREMENT cohort investigator group]), and higher organism MICs for meropenem have been associated with negative clinical outcomes for CRE. However, many of these studies were limited either by sample size, source of infection, or organism type or did not analyze a totality of patient risk factors for CRE (14–18). Furthermore, updated evidence on risk factors or protective factors that encompass all CRE infection sources are warranted with newer BL/BLI treatments, especially around negative outcomes for CRE.

We aimed to identify specific variables involved in CRE treatment that are associated with clinical failure. We sought to determine these factors by comparing patient cohorts of each outcome among CRE infections and by performing multivariable logistic regression using stepwise selection to identify factors with significant associations.

## RESULTS

There were 880 total Gram-negative infections screened in the collected Gram-negative retrospective database, which was filtered to 588 Gram-negative infections from 2010 to 2020 among 2 medical centers. There were then 170 infections preliminarily identified as CRE, and this list was narrowed to 140 confirmed CRE infections by CLSI carbapenem-resistant MIC. Table 1 details patient characteristics separated by clinical success/failure for variables including at least 10% of CRE cases. Variables were separated into demographics, multidrug resistance (MDR) risk factors (health care-associated pneumonia from 2005 American Thoracic Society Infectious Diseases Society of America [ATS IDSA] guidelines), other infection and treatment categories, and outcome (19). The number of applicable cases was noted with [*n* =] for variables that were not available for all cases. A list of all variables tested, not only those with at least 10% variability, is included in Table S1 in the supplemental material.

**TABLE 1:** Case characteristics by clinical outcome

Characteristic^*a*^	No. of CRE cases (*n* = 140)^*b*^			*P* value^*c*^
	Total (*n* = 140)	Clinical failure (*n* = 55)	Clinical success (*n* = 85)	
Demographics				
Institution, 1	78 (56)	29 (53)	49 (58)	0.57
Age (yrs)	60.9 ± 17	59.4 ± 19	61.8 ± 15	0.41
Sex, male	80 (57)	29 (53)	51 (60)	0.40
Race				
African-American	80 (57)	33 (60)	47 (55)	0.58
Caucasian	51 (36)	19 (35)	32 (38)	0.71
BMI (kg/m^2^) (median [IQR])	27.7 (12)	28.6 (12)	26.8 (11)	0.20
Obesity (BMI ≥ 30)	50 (36)	23 (42)	27 (32)	0.23
Charlson comorbidity index	5.2 ± 3.0	4.9 ± 2.8	5.3 ± 3.2	0.41
Comorbid characteristics				
Chronic heart failure	23 (16)	11 (20)	12 (14)	0.36
Peripheral vascular disease	28 (20)	7 (13)	21 (25)	0.08*
Stroke/TIA	28 (20)	11 (20)	17 (20)	1.0
Dementia	15 (11)	6 (11)	9 (11)	0.95
COPD	27 (19)	12 (22)	15 (18)	0.54
Connective tissue disease	16 (11)	3 (5.5)	13 (15)	0.07*
Diabetes w/o end organ damage	21 (15)	9 (16)	12 (14)	0.72
Diabetes w/end organ damage	37 (26)	15 (27)	22 (26)	0.86
Diabetes mellitus, any	58 (41)	24 (44)	34 (40)	0.67
Moderate/severe CKD/dialysis	46 (33)	20 (36)	26 (31)	0.48
Chronic dialysis	16 (11)	12 (22)	4 (4.7)	<0.01*
Tumor	18 (13)	6 (11)	12 (14)	0.58
Immunocompromised (APACHE-defined)*	11 (7.9)	7 (13)	4 (4.7)	0.11
MDR risk factors and severity				
Admitted from:				
Home	68 (49)	25 (46)	43 (51)	0.55
NH, SNF, LTCF	41 (29)	16 (29)	25 (29)	0.97
Outside hospital	21 (15)	11 (20)	10 (12)	0.18
MDR risk factors	2.4 ± 1.3	2.5 ± 1.5	2.3 ± 1.3	0.25
Prior surgery in 30 days before index culture	23 ± 16	7 ± 13	16 ± 19	0.36
Prior hosp ≥48 h in 90 days before index culture	99 ± 71	40 ± 73	59 ± 69	0.67
Prior abx ≥24 h in 90 days before index culture	103 ± 74	44 ± 80	59 ± 69	0.17
Prior resistant organism infection in 365 days before index culture	45 ± 32	18 ± 33	27 ± 32	0.91
APACHE II	22.1 ± 8.8	24.4 ± 8.8	20.6 ± 8.6	0.01*
Pitt bacteremia score (0 to 14 score)	3.0 ± 2.4	3.5 ± 2.5	2.6 ± 2.3	<0.05*
SOFA score	5.7 ± 3.8	7.1 ± 4.1	4.7 ± 3.3	<0.01*
SIRS criteria, meeting	122 (87)	50 (91)	72 ± 85	0.28
Admitted to ICU	113 (80)	48 (87)	64 (75)	0.08*
Source				
Primary bacteremia	19 (14)	10 (18)	9 (11)	0.20
IAI	23 (16)	6 (11)	17 (20)	0.16
Respiratory*	53 (38)	24 (44)	29 (34)	0.26
UTI	28 (20)	9 (16)	19 (22)	0.39
Other^*d*^	17 (12)	6 (11)	11 (13)	0.72
CRE organism				
Escherichia coli	23 (16)	5 (9.1)	18 (21)	0.06*
Klebsiella pneumoniae	93 (66)	42 (76)	51 (60)	<0.05*
Carbapenemase production (*n* = 139)				
Not tested	74 (53)	25 (46)	49 (58)	0.19
Carbapenemase detected, any	47 (34)	20 (37)	27 (32)	0.52
KPC	45 (32)	19 (35)	26 (31)	0.57
None found	18 (13)	9 (17)	9 (11)	0.30
All organisms				
Escherichia coli	27 (19)	6 (11)	21 (25)	0.04*
Klebsiella pneumoniae	94 (67)	43 (78)	51 (60)	0.03*
Pseudomonas aeruginosa	25 (18)	8 (15)	17 (20)	0.41
Infection characteristics				
No. of organisms in index culture	1.7 ± 0.84	1.5 ± 0.72	1.8 ± 0.90	<0.05*
Polymicrobial (>1 in index culture)	69 (49)	22 (40)	47 (55)	0.08*
ID consult*	138 (99)	54 (98)	84 (99)	1.0
Surgical consult	27 (20)	10 (18)	17 (21)	0.74
Surgical source control procedure (choice = any)^*e*^	24 (17)	8 (15)	16 (19)	0.51
Antibiotic agents				
BL/BLI (CZA, C/T, M-V)	117 (84)	47 (86)	70 (82)	0.63
CZA	76 (54)	29 (53)	47 (55)	0.77
M-V	45 (32)	19 (35)	26 (31)	0.62
Polymyxin, colistin, or aminoglycoside	54 (39)	27 (49)	27 (32)	0.04*
Polymyxin/colistin	22 (16)	12 (22)	10 (12)	0.11
Aminoglycoside (any)	37 (26)	18 (33)	19 (22)	0.17
Tigecycline	24 (17)	10 (18)	14 (17)	0.79
Carbapenem (any)	67 (48)	27 (49)	40 (47)	0.81
Ertapenem	15 (11)	5 (9.1)	10 (12)	0.62
Meropenem	58 (41.4)	23 (41.8)	35 (41.2)	0.94
Extended infusion (*n* = 54)	24 (44)	12 (57)	12 (36)	0.13
CRE MICs and active therapy				
Meropenem MICs* (median [Q_1_ to Q_3_]) (*n* = 137)^*f*^^,^^*g*^	16 (4.0 to 16)	16 (4.0 to 16)	8.0 (2.0 to 16)	0.16
Meropenem resistant, MIC ≥ 4 or R (*n* = 137)^*g*^	104 (76)	43 (80)	61 (74)	0.41
Imipenem resistant, MIC ≥ 4 or R (*n* = 57)^*g*^	44 (79)	18 (86)	26 (74)	0.50
Ertapenem resistant, MIC ≥ 2 or R (*n* = 139)^*g*^	138 (99)	54 (100)	84 (99)	1.0
CZA resistant, MIC ≥ 16/4 or R (*n* = 102)^*g*^	7 (6.9)	5 (11)	2 (3.6)	0.24
M-V resistant, MIC ≥ 16/8 or R (*n* = 38)^*g*^	1 (2.6)	1 (5.9)	0	0.45
Active therapy given^*h*^	131 (94)	51 (93)	80 (94)	0.74
Time to active abx (days) (*n* = 132)	3.2 ± 4.5	2.8 ± 2.0	3.4 ± 5.5	0.48
Median (IQR)	3.0 (3.0)	3.0 (3.0)	2.0 (3.0)	0.60
Range	−4.0 to 37	0 to 8.0	−4.0 to 37	
Within 48 h from index culture	63 (48)	22 (43)	41 (51)	0.37
Outcomes				
Clinical failure	55 (39)	55 (100)	0	
Mortality, 30-day (index culture)	29 (21)	29 (53)	0	**<0.01**
Recurrence, 30-day (on or w/in 30 days from end of tx)	12 (8.6)	12 (22)	0	**<0.01**
Symptom resolution	106 (75)	25 (46)	80 (94)	**<0.01**
Length of stay (days) (median [IQR])	25.5 (34)	34.0 (37)	22.0 (31)	**0.04**
Adverse drug reaction				
Nephrotoxicity	14 (10)	5 (9.1)	9 (11)	0.77
C. difficile	3 (2.1)	2 (3.6)	1 (1.2)	0.56
Neutropenia	2 (1.4)	1 (1.8)	1 (1.2)	1.0
None	119 (85)	47 (86)	72 (85)	0.90

a CRE, carbapenem-resistant *Enterobacterales*; IQR, interquartile range; BMI, body mass index; COPD, chronic obstructive pulmonary disorder; w/o, without; w/, with; CKD, chronic kidney disease; immunocompromised (APACHE-defined), any chemo or radiation therapy within 30 days, HIV/AIDS with CD4 of <200, or chronic steroids (equivalent to >40 mg prednisone); APACHE (II-score), Acute Physiology and Chronic Health Evaluation (II score); MDR, multidrug resistant; NH, nursing home; SNF, skilled nursing facility; LTCF, long-term care facility; hosp, hospitalization; abx, antibiotics; SOFA, Sequential Organ Failure Assessment; SIRS, Systemic Inflammatory Response Syndrome; ICU, intensive care unit; IAI, intra-abdominal infection; UTI, urinary tract infection; ID, infectious diseases; BL/BLI, beta-lactam/beta-lactamase inhibitor; CZA, ceftazidime-avibactam; C/T, ceftolozane-tazobactam; M-V, meropenem-vaborbactam; C. difficile, Clostridioides difficile infection; neutropenia, absolute neutrophil count (ANC) of <1,500 cells/mm^3^ or a 50% decrease if ANC is <1,500 cells/mm^3^ at baseline. Institution, 1, refers to the variable of 'institution' which hospital system contained the cases among the two institutions. Institution, 1, refers to one system where the majority of cases came from in comparison to Institution 2. *, indicates the variable was either predefined [characteristic column] or met the ≤0.1 cutoff [*P* value column] for entry into stepwise regression.

b Values are *n* (%) or mean ± SD unless otherwise indicated.

c Bold values indicate significance at a *P* value of <0.05.

d Sources were skin and soft tissue infection (SSTI), central line-associated bloodstream infection (CLABSI), bone and joint infections, invasive prosthetic device infections, and unknown sources.

e Surgical source control procedures included intravenous catheter removal, valvular repair/replacement, invasive device removal, incision and drainage, drain placement, debridement, resection, excision, or amputation.

f Ordinal MICs were calculated using inference of MICs where an MIC of ≤0.25 = 0.25, ≤0.5 = 0.5, <1 = 0.5, <4 = 2, >1 = 2, >8 = 16, ≥16 = 16, and >16 = 32.

g Available *in vitro* CRE MICs (automated system, Etest, Kirby Bauer, or broth microdilution methods).

h Active therapy defined as an antibiotic given for the index infection that is nonresistant by *in vitro* organism MIC.

Cases were divided relatively evenly between the two medical centers (56% and 54%, respectively). Among the 140 cases, there were 139 patients, with 1 patient having a repeat case outside the 60-day exclusion window. The mean age for all CRE cases was 61 years, and they were mostly male (57%), were mostly African-American (57%), had a moderate mean body mass index (BMI) of 27.7 kg/m^2^, and had a mean Charlson comorbidity index of 5.2. Infection severity by mean APACHE-II score was 22.1, SOFA was 5.7, and most patients (87%) met systemic inflammatory response syndrome (SIRS) criteria and were admitted to the intensive care unit (ICU; 80%). The primary source of CRE was respiratory (38%), with Klebsiella pneumoniae (67%) as the majority pathogen. Novel BL/BLI antibiotics (84%) were primarily used followed by carbapenems (48%) and polymyxins or aminoglycosides (39%).

Clinical failure occurred in 39% of cases. Secondary outcomes included all-cause 30-day mortality at 21% and achieving symptom resolution in 75% of cases. Thirty-day recurrence was low at 8.6%. All secondary outcomes were significantly different between clinical success and clinical failure groups. Overall, adverse events were low, with 85% of patients having no adverse drug reaction. Of the adverse events reported, nephrotoxicity (10%) was the most common followed by Clostridioides difficile infection (2.1%) and neutropenia (1.4%).

**FIG 1:**
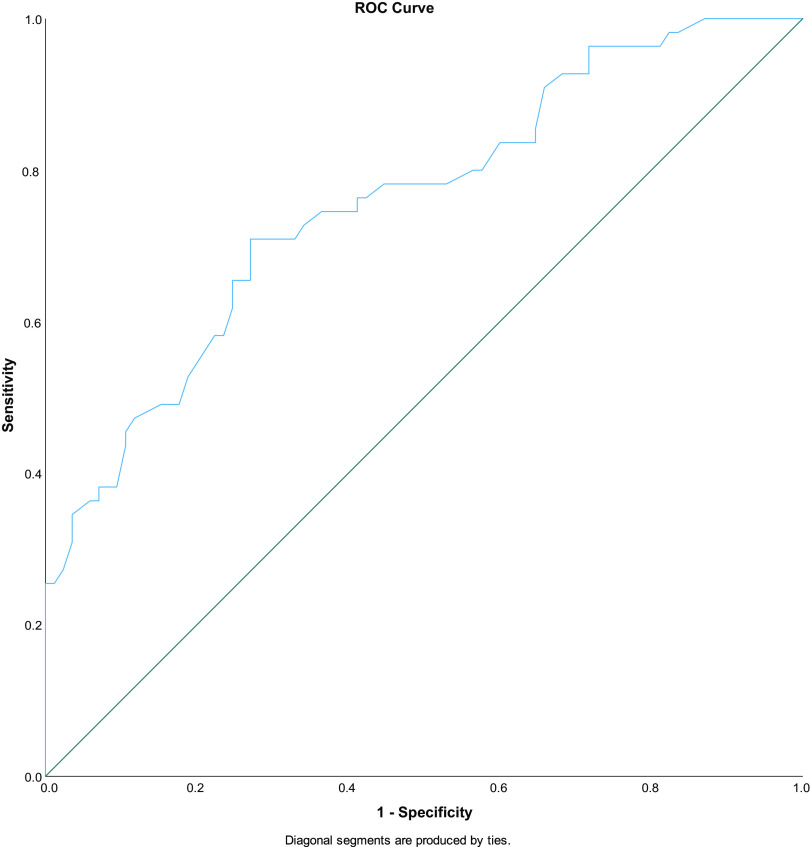
AUC/ROC curve for multivariable regression model—clinical failure.

There were 14 variables with significant differences between clinical success and failure that met our *P* value threshold cutoff, along with the 5 prespecified additional variables, for entry into stepwise selection for the multivariable regression model. Five colinear variables were removed, and the remaining variables underwent stepwise selection (see Tables S2 to S4 in the supplemental material). The final five stepwise-selected variables made up the multivariable regression model for predictors of clinical failure which was rerun to include all CRE cases (Table 2 and 3). Chronic dialysis as a comorbidity (adjusted odds ratio [aOR], 5.86; 95% confidence interval [CI], 1.51 to 22.7), each unit of SOFA score (aOR, 1.18; 95% CI, 1.06 to 1.32), and Klebsiella pneumoniae in index culture (aOR, 3.09; 95% CI, 1.28 to 7.47) were significantly associated predictors with clinical failure compared with clinical success. No significant joint outliers were found between standard residual plots against Cook’s distance and against leverage values (see Fig. S1 and S2 in the supplemental material). A receiver operating characteristic (ROC) curve was also done to illustrate the model’s predictive sensitivity and specificity for CRE cases of clinical success and failure (Fig. 1).

**TABLE 2:** Multivariable logistic regression^*a*^—predictors of clinical failure

Variable	Odds ratio (95% CI)	Adjusted odds ratio (95% CI)	*P* value^*b*^
Comorbid: chronic dialysis	5.65 (1.72–18.6)	5.86 (1.51–22.7)	0.01
SOFA score	1.20 (1.08–1.33)	1.18 (1.06–1.32)	<0.01
Organism: Klebsiella pneumoniae	2.39 (1.10–5.18)	3.09 (1.28–7.47)	0.01
Comorbid: connective tissue disease	0.320 (0.087–1.18)	0.279 (0.066–1.17)	0.08
No. of concomitant organisms	0.635 (0.404–0.998)	0.633 (0.380–1.06)	0.08

a Stepwise logistic regression had entry at significance of 0.05 and removal at significance of 0.1. The following nine variables were included in the stepwise selection process for regression due to meeting univariate ≤0.1 *P* value and 10% variability: comorbid: peripheral vascular disease; comorbid: connective tissue disease (OA, RA); comorbid: chronic dialysis; admission to ICU; SOFA score; organism: Escherichia coli; organism: Klebsiella pneumoniae; no. of concomitant organisms; and Abx, colistin/polymyxin/aminoglycoside. The following five predefined variables were also included in stepwise selection process: source: respiratory, immunocompromised status (APACHE-defined), meropenem MICs, time (days), and ID consult. Nine variables were removed due to backward selection when significance was ≥0.1. The constant was 0.216 with a *P* value of 0.02.

b *P* values represent adjusted odds ratios.

**TABLE 3:** Model summary statistics^*a*^

Parameter	Data
Pseudo R-square	
Cox & Snell	0.218
Nagelkerke	0.295
AUC/ROC (mean ± SE)	75.8 ± 0.042
Classification table: overall correct	70%

a No dual outliers (Fig. S1 and S2) were present.

Other hypotheses about CRE infections were tested using the developed multivariable regression model. Neither immunocompromised status; respiratory source of infection; primary bacteremia source; any novel BL/BLI antibiotic; use of polymyxin, colistin, or aminoglycoside antibiotic; ID consult; meropenem MIC; nor time were statistically associated with clinical failure on multivariable adjustment (Table 4).

**TABLE 4:** Clinical hypotheses tested against multivariable model for clinical failure

Tested variable^*a*^	Odds ratio (95% CI)	Adjusted odds ratio (95% CI)	*P* value^*b*^
Immunocompromised status (APACHE)	2.95 (0.822–10.6)	2.34 (0.533–10.3)	0.26
Source: respiratory	1.50 (0.745–3.00)	1.05 (0.444–2.50)	0.91
Source: primary bacteremia	1.88 (0.709–4.97)	1.94 (0.581–6.49)	0.28
Novel BL/BLI	1.26 (0.495–3.20)	1.16 (0.396–3.40)	0.79
Polymyxin, colistin, or aminoglycoside	2.07 (1.03–4.17)	1.62 (0.706–3.71)	0.26
ID consult	0.643 (0.039–10.5)	0.517 (0.027–9.86)	0.66
Meropenem MICs (*n* = 137)	1.04 (0.988–1.09)	1.01 (0.952–1.08)	0.71
Time (days)	1.000 (0.999–1.000)	1.000 (0.999–1.001)	0.96

a Each “tested variable” was adjusted against all variables from Table 2. Immunocompromised (APACHE-defined), any chemo or radiation therapy within 30 days, HIV/AIDS with CD4 of <200, or chronic steroids (equivalent to >40 mg prednisone); BL/BLI, beta-lactam/beta-lactamase inhibitor; ID, infectious diseases.

b *P* values represent adjusted odds ratios.

## DISCUSSION

Patients with CRE infections among this cohort had severe infections and presented very ill given that 80% were admitted to the ICU and had substantially elevated severity scores that would be associated with roughly 20% to 40% mortality according to SOFA and APACHE-II estimates. Patients with these infections also had numerous comorbidities, with an average number of comorbidities of >5 from the Charlson comorbidity list, which may have contributed to the acquisition of a CRE. It is also telling that most patients had significant health care contact, with 71% having a prior hospitalization for ≥48 h in the past 90 days and 74% having received antibiotics in the past 90 days. The data are consistent with known literature on CRE, where these organisms are likely to occur in older, previously antibiotic-exposed, severely ill patients who have frequent contact with the health care system.(10, 18, 20, 21).

Notably, the severity measurement of SOFA score was most statistically associated with clinical failure. This finding intuitively makes sense given that the most severely presenting patients have the lowest likelihood of a positive outcome. This finding also shows that CRE infections must be treated aggressively before patients present with critical illness to ensure effective cure. Chronic dialysis was also found to be a strong predictive variable of clinical failure, with these patients being almost six times more likely to have clinical failure than nondialysis patients after multivariable adjustment. This finding is also biologically plausible given a dialysis patient’s poor drug clearance, more frequent health care contact, and less studied dosing parameters that may lead to underdosed antibiotics. Klebsiella pneumoniae as an index culture organism was also an associated statistical predictor of clinical failure, and this result should lead clinicians to treat CRE caused by Klebsiella with special concern. Klebsiella bacteria may have more potent beta-lactamases or other microbiological signal mechanisms that render them more evasive of antibiotics and more virulent than other CRE organisms (18, 22, 23). Interim data from a recent CRE matched multinational European study also support Klebsiella pneumoniae having higher disease virulence that is associated with higher mortality (24). These combined significant predictors suggest that patients with CRE who are on chronic dialysis, have elevated SOFA scores, or grow Klebsiella pneumoniae in index cultures should be prioritized for aggressive treatment, as they are the most associated with poor clinical outcomes. These significant patient risk factors can potentially be used to initiate broader or earlier access to restricted/stewarded antimicrobials to avert a poor clinical outcome. It also suggests that more research is required into these variables to understand the specific underlying mechanisms leading to larger clinical failure.

The number of concomitant organisms in index culture was also found as a negative predictor of clinical failure, but this result was not statistically significant. This finding may be due to patients with multiple organisms having a higher likelihood to have either a more chronic infection or a lower severity of illness that is then associated with better outcomes. The last variable selected in the regression was the comorbidity of connective tissue disease which was a predictor against clinical failure. This result may be an artifact of data collected and multiple variables tested, given that this variable was not found significant. The biological plausibility for this variable to lessen CRE clinical failure is also unlikely with examples of connective tissue disease, including osteoarthritis or rheumatoid arthritis, not supported by any current data.

When testing certain clinical hypotheses against the developed multivariable model, we found that none reached statistical significance (Table 4). Therefore, the current data sample is unable to suggest that immunocompromised status, a respiratory source of infection, primary bacteremia as a source of infection, any novel BL/BLI use, any polymyxin/colistin/aminoglycoside use, ID consult, meropenem MICs, or time has an association with clinical failure or success for CRE. While the current clinical sample was unable to reject any null hypothesis, the associated odds ratio after adjustment may offer insight into which hypotheses may warrant further investigation. Immunocompromised status was adjusted downward, but its large association and biological plausibility for poor outcomes lend it toward further investigation as a potential risk factor with a larger sample size. While respiratory source seems unlikely as an influential variable given its strong adjustment downward toward 1.0 in our study, contrasting with the results from Shields et al. (14), primary bacteremia may be more likely given the higher adjustment toward 2.0. Individual antibiotics or their use as a class likely requires larger sample sizes to detect differences between outcomes. The limited number of CRE cases without an ID consult may have affected the ID consult-specific variable to not detect a difference. Time as a variable (in days, by admission date) was also not significant and was removed in the final clinical failure model, with one explanation possibly being lower CRE cases from admissions prior to 2016. Existing literature for improved mortality over time references mainly recent carbapenem-resistant Pseudomonas infections over other Gram-negative infections, which may partially explain why no difference was found in these only-*Enterobacterales* infections.(4).

The study’s strengths are the wide variety of CRE cases that were sampled across a time period of 2010 to 2020 in a specific urban region. The number of CRE cases were also considerable, and data were identified for the vast majority of variables. Another strength of the study is the predefined methodologies, definitions, and analysis for the outcome of clinical failure. An attempt to account for data among established literature in the statistical analysis was made with predefined variables for inclusion into the stepwise regression process, instead of relying only on inductive univariate *P* value cutoffs. An aggressive univariate *P* value cutoff of ≤0.1 was chosen for variable inclusion in stepwise selection. In addition, a 10% cutoff for variability was made in order to remove spurious data that may occur due to low numbers of cases for a variable. These methodologic choices can ensure that only variables that significantly and broadly affect CRE case outcomes are chosen for analysis. The final regression model was also tested for outliers using residual plots, and standard measures of model summary statistics, such as pseudo-R square (Cox and Snell, Nagelkerke), classification assignment, and area under the receiver operating characteristic curve, were provided for the final model with an area under the concentration-time curve (AUC)/ROC of 75.8, indicating acceptable discrimination.

However, this study must be interpreted in regard to its limitations. Notably, this research is a retrospective observational study, meaning that random allocation was not done and selection bias can inherently exist between groups. Composite endpoints can also introduce bias if a component diverges from another, is irrelevant to clinical practice, or is significantly buoyed by one major outcome. The inclusion of repeat patient cases of CRE, even only one patient, outside the 60-day exclusion window may have biased the variable modeling with repeated characteristics. Only 47% of CREs were evaluated for carbapenemases, and Klebsiella pneumoniae carbapenemase (KPC) was the only specific carbapenemase isolated, which can limit external validity to other carbapenemase epidemiology. The majority of CRE cases also came from respiratory or urinary sources, which can have diagnostic difficulty compared with other infection sources. The sample size also offers another limitation, especially for certain variables and outcomes that occurred in a smaller portion of cases. Small sample sizes may have contributed to not detecting an association for some of the predefined hypotheses. In contrast, the heterogeneity of CRE types across many different sources of infection may have complicated efforts to detect significant results. Also, while a univariate cutoff of 0.1 can guard against variables that may coincidentally meet criteria and have statistical but not clinical significance, the aggressive cutoff may have excluded clinically significant variables that did not meet statistical significance. An attempt to incorporate variables from literature may not have captured every clinically significant variable for each outcome. In the future, a predefined directed acyclic graph could be created as a consensus among the research group to identify potential confounders using causal inference for these outcomes and to appropriately account for clinical variables.

In conclusion, the manuscript adds clinical focus to specific characteristics of CRE infections that are associated with the poorest clinical outcomes. Severity scores, such as the SOFA score, can be used to effectively predict a CRE patients’ clinical outcome. Special attention should be paid to Klebsiella pneumoniae, as it was associated with higher clinical failures than other organisms. Chronic dialysis may also be an area for further investigation with antibiotic dosing to improve outcomes among CRE infections due to its especially high association with clinical failure. Rapid diagnostics and aggressive antibiotic use early in disease may be warranted for these high-risk characteristics among CRE infections, with recent research in CRE bacteremia showing positive outcomes (25). Research should continue on carbapenem-resistant *Enterobacterales* infections in large, randomized cohorts and case-control studies to further identify best practices to promote treatment success.

## MATERIALS AND METHODS

We conducted a retrospective, observational, cohort study of patients admitted with a CRE infection and sampled from 2010 to 2020 at 2 medical systems in Detroit, Michigan. Patient demographics, medical history, risk factors for multidrug resistance (MDR) acquisition (e.g., prior antibiotic and health care exposures), clinical laboratory data, disease severity (APACHE-II score, SOFA score, Pitt bacteremia score, and SIRS criteria), microbiology, infection source, treatment parameters (antibiotic, dose, duration, and time to active therapy), and outcomes were collected from the electronic medical record. Patients were included if they were ≥18 years old and culture positive for an organism in the *Enterobacterales* order causing clinical infection with resistance to at least one carbapenem defined by Clinical and Laboratory Standards Institute (CLSI) breakpoints 31st edition (26). Patients were excluded if they were prisoners, were pregnant, or had prior patient encounters with CRE infections within 60 days.

This study does not include factors necessitating patient consent, as approved by the institutional review board (IRB). Furthermore, the design and reporting of this study have been approved by local institutional review boards.

### Definitions.

Carbapenem resistance was defined as any carbapenem MIC at or above the resistance breakpoint for *Enterobacterales* according to CLSI, which are an MIC of ≥2 μg/mL for ertapenem or ≥4 μg/mL for imipenem or meropenem.(26) In contrast, carbapenem resistance according to EUCAST has MICs of >0.5 for ertapenem, >4 for imipenem, and >8 for meropenem (27). Microbiological MIC was determined by automated susceptibility (BD Phoenix and Vitek 2), Etest, or broth microdilution, by whichever method was reported among patient cases. The primary outcome target was clinical failure defined as a composite of either 30-day mortality, 30-day microbiologic recurrence, or worsening/failure to improve clinically throughout antibiotic treatment. Thirty-day mortality was measured from index culture collection, and 30-day microbiologic recurrence was measured from either index culture or the end of antibiotic treatment, whichever occurred later. Secondary outcomes included individual components of the primary outcome, adverse drug reactions, and symptom resolution defined as a composite endpoint of either (i) no worsening/failure to improve clinically and reduction/resolution in white blood cell (WBC) count or fevers or (ii) afebrile with no leukocytosis over 24 h at index culture collection and no worsening/failure to improve clinically during antibiotic therapy.

### Statistical analysis.

Nominal variables were compared using Pearson chi-square test or Fisher’s exact test, as appropriate. Ordinal and continuous variables were analyzed using the Mann-Whitney U test and Student’s *t* test for nonparametric and parametric data, respectively. Multivariable logistic regression was utilized to determine risk factors associated with clinical failure among all CRE cases. Patient cohorts of clinical failure and clinical success were compared by each variable. Predictor variables regarding clinical failure required at least 10% variability (variable covering at least 10% of total cases) to be eligible for entry into the stepwise selection regression model. Variables also needed a *P* value significance of ≤0.10 on univariate analysis for entry. Additional prespecified variables were entered into stepwise selection regardless of the *P* value cutoff as random effect variables, due to their basis in literature to affect CRE outcomes and/or their biological plausibility. These variables included respiratory source of infection, immunocompromised status, ID consult, and meropenem MICs (14–18, 28, 29). Time (function of admission date) was also entered as a variable in stepwise selection to account for the possibility that treatments and the care of patients have naturally improved over time (4).

The multivariable stepwise logistic regression model followed backward likelihood ratio test stepwise selection with a cutoff of 0.1. Variance inflation factors (VIFs) determined whether collinearity between variables was present. If variables qualified for the stepwise selection regression process and had VIFs of >2, the colinear variables were isolated and underwent stepwise selection to eliminate collinearity by including only the resultant variable(s). Final regression models for each outcome had predictor variables and odds ratios reported with 95% confidence intervals, pseudo-R square values listed, and a receiver operating curve (ROC) area under the concentration-time curve (AUC) classification graph to illustrate how well the model predicted the clinical failure outcome. Outliers for each model were identified by plotting studentized residuals against each regression model’s leverage points and Cook’s distance values. Significant outliers identified as visually asymmetric over the standard residual and having high Cook’s influence or leverage value on both plots were removed, and the regression model was run again after removal. Statistics were calculated using SPSS Statistics, version 28.0 (IBM Corp., Armonk, NY).

### Data availability.

Patient clinical data were collected and managed using the Research Electronic Data Capture (REDCap) tool hosted at Wayne State University (30). The data and code used for the analysis are available on request.
