# Supplementary material for: Risk Factors for Carbapenem-Resistant Enterobacterales Clinical Treatment Failure
Source: Microbiol Spectr. 2023 Jan 9;11(1):e02647-22. doi: 10.1128/spectrum.02647-22 (PMC9927167; doi:10.1128/spectrum.02647-22)

Table S1. Case Characteristics by Clinical Outcome, Full

**Characteristic**	**CRE Cases (N=140)**			**p-value**
	Total (n=140)	Clinical Failure (n=55)	Clinical Success (n=85)	**p-value**
Demographics				
Institution, 1 (%)	78 (55.7)	29 (52.7)	49 (57.6)	0.57
Age, years; mean, (SD)	60.9 (16.5)	59.4 (18.5)	61.8 (15)	0.41
Sex, male	80 (57.1)	29 (52.7)	51 (60.0)	0.40
Race African-American	80 (57.1)	33 (60)	47 (55.3)	0.58
Caucasian	51 (36.4)	19 (34.5)	32 (37.6)	0.71
Hispanic	1 (0.7)	1 (1.8)	0	0.39
Other	8 (5.7)	2 (3.6)	6 (7.1)	0.48
BMI, kg/m^2; median, (IQR)	27.7 (12.1)	28.6 (12.3)	26.8 (10.8)	0.20
Obesity (BMI≥30)	50 (35.7)	23 (41.8)	27 (31.8)	0.23
Charlson comorbidity index (CCI); mean, (SD)	5.2 (3.0)	4.9 (2.8)	5.3 (3.2)	0.41
Comorbid: Myocardial Infarction	9 (6.4)	2 (3.6)	7 (8.2)	0.48
Comorbid: Chronic Heart Failure	23 (16.4)	11 (20.0)	12 (14.1)	0.36
Comorbid: Peripheral vascular disease	28 (20.0)	7 (12.7)	21 (24.7)	0.08*
Comorbid: Stroke/TIA	28 (20.0)	11 (20.0)	17 (20.0)	1.0
Comorbid: Dementia	15 (10.7)	6 (10.9)	9 (10.6)	0.95
Comorbid: COPD	27 (19.3)	12 (21.8)	15 (17.6)	0.54
Comorbid: Asthma	8 (5.7)	4 (7.3)	4 (4.7)	0.71
Comorbid: Connective tissue disease	16 (11.4)	3 (5.5)	13 (15.3)	0.07*
Comorbid: PUD (not GERD)	4 (2.9)	1 (1.8)	3 (3.5)	1.0
Comorbid: Chronic hepatitis w/o cirrhosis	8 (5.7)	5 (9.1)	3 (3.5)	0.26
Comorbid: Moderate/severe liver disease	9 (6.4)	3 (5.5)	6 (7.1)	1.0
Comorbid: Diabetes w/o end organ damage	21 (15)	9 (16)	12 (14)	0.72
Comorbid: Diabetes w/end organ damage	37 (26)	15 (27)	22 (26)	0.86
Comorbid: Diabetes mellitus, any	*58 (41.4)*	*24 (43.6)*	*34 (40.0)*	*0.67*
Comorbid: Hemiplegia	7 (5.0)	2 (3.6)	5 (5.9)	0.70
Comorbid: Moderate/severe CKD/dialysis	46 (32.9)	20 (36.4)	26 (30.6)	0.48
Comorbid: Chronic dialysis	16 (11.4)	12 (21.8)	4 (4.7)	**<0.01***
Comorbid: Tumor w/o mets	9 (6.4)	5 (9.1)	4 (4.7)	0.32
Comorbid: Tumor w/mets	9 (6.4)	1 (1.8)	8 (9.4)	0.09
Comorbid: Tumor (all)	*18 (12.9)*	*6 (10.9)*	*12 (14.1)*	*0.58*
Comorbid: Leukemia	2 (1.4)	1 (1.8)	1 (1.2)	1.0
Comorbid: Lymphoma	0	0	0	-
Comorbid: AIDS (CD4<200)	1 (0.7)	1 (1.8)	0	0.39
Comorbid: HIV	2 (1.4)	1 (1.8)	1 (1.2)	1.0
Comorbid: PWID	9 (6.4)	4 (7.3)	5 (5.9)	0.74
Comorbid: Cystic Fibrosis	0	0	0	-
Comorbid: None	9 (6.4)	2 (3.6)	7 (8.2)	0.48
Immunocompromised (APACHE-defined), (%)	11 (7.9)	7 (12.7)	4 (4.7)	0.11
MDR Risk Factors and Severity				
Admitted from, n (%)				
Home	68 (48.6)	25 (45.5)	43 (50.6)	0.55
NH, SNF, LTCF	41 (29)	16 (29)	25 (29)	0.97
Transfer from outside hospital	21 (15)	11 (20)	10 (12)	0.18
MDR Risk factors, number; mean, (SD)	2.4 (1.3)	2.5 (1.5)	2.3 (1.3)	0.25
Prior surgery in 30 days before index culture	23 (16.4)	7 (12.7)	16 (18.8)	0.34
Prior hosp ≥48h in 90 days before index culture	99 (70.7)	40 (72.7)	59 (69.4)	0.67
Prior abx ≥24h in 90 days before index culture	103 (73.6)	44 (80.0)	59 (69.4)	0.17
Prior resistant organism infection in 365 days before index culture	45 (32.1)	18 (32.7)	27 (31.8)	0.91
None	12 (8.6)	5 (9.1)	7 (8.2)	1.0
APACHE II; mean, (SD)	22.1 (8.8)	24.4 (8.8)	20.6 (8.6)	**0.01***
Pitt Bacteremia Score [0-14 score]; mean, (SD)	3.0 (2.4)	3.5 (2.5)	2.6 (2.3)	**<0.05***

SOFA Score; mean, (SD)	5.7 (3.8)	7.1 (4.1)	4.7 (3.3)	**<0.01***
SIRS Criteria, meeting; n (%)	122 (87.1)	50 (90.9)	72 (84.7)	0.28
Admitted to ICU, n (%)	112 (80.0)	48 (87.3)	64 (75.3)	0.08*
Source, n (%)				
Source: Primary Bacteremia	19 (13.6)	10 (18.2)	9 (10.6)	0.20
Source: Bone/Joint	1 (0.7)	0	1 (1.2)	1.0
Source: IAI	23 (16.4)	6 (10.9)	17 (20.0)	0.16
Source: Respiratory	53 (37.9)	24 (43.6)	29 (34.1)	0.26
Source: SSTI	11 (7.9)	5 (9.1)	6 (7.1)	0.75
Source: UTI	28 (20.0)	9 (16.4)	19 (22.4)	0.39
Source: Invasive prosthetic	1 (0.7)	0	1 (1.2)	1.0
Source: CLABSI	2 (1.4)	1 (1.8)	1 (1.2)	1.0
Source: Other	2 (1.4)	0	2 (2.4)	0.52
CRE Organism, n (%)				
Citrobacter freundii	3 (2.1)	1 (1.8)	2 (2.4)	1.0
Enterobacter aerogenes	5 (3.6)	2 (3.6)	3 (3.5)	1.0
Enterobacter cloacae	14 (10)	3 (5.5)	11 (13)	0.15
Escherichia coli	23 (16)	5 (9.1)	18 (21)	0.06*
Klebsiella oxytoca	2 (1.4)	1 (1.8)	1 (1.2)	1.0
Klebsiella pneumoniae	93 (66)	42 (76)	51 (60)	<**0.05***
Morganella morganii	0	0	0	-
Proteus mirabilis	0	0	0	-
Proteus vulgaris	0	0	0	-
Providencia stuartii	0	0	0	-
Raoultella ornithinolytica	1 (0.7)	0	1 (1.2)	1.0
Serratia marcescens	4 (2.9)	2 (3.6)	2 (2.4)	0.65
Carbapenemase-production, n (%); (n=139)				
Not tested	74 (53)	25 (46)	49 (58)	0.19
Carbapenemase detected, any	47 (34)	20 (37)	27 (32)	0.52
KPC	45 (32)	19 (35)	26 (31)	0.57
VIM	0	0	0	-
IMP	0	0	0	-
OXA	0	0	0	-
NDM	2 (1.4)	1 (1.9)	1 (1.2)	1.0
None found	18 (13)	9 (17)	9 (11)	0.30
All Organisms, n (%)				
Acinetobacter baumanii	5 (3.6)	1 (1.8)	4 (4.7)	0.65
Citrobacter freundii	3 (2.1)	1 (1.8)	2 (2.4)	1.0
Enterobacter aerogenes	6 (4.3)	2 (3.6)	4 (4.7)	1.0
Enterobacter cloacae	14 (10)	3 (5.5)	11 (13)	0.15
Escherichia coli	27 (19)	6 (11)	21 (25)	**0.04***
Klebsiella oxytoca	2 (1.4)	1 (1.8)	1 (1.2)	1.0
Klebsiella pneumoniae	94 (67)	43 (78)	51 (60)	**0.03***
Morganella morganii	3 (2.1)	1 (1.8)	2 (2.4)	1.0
Proteus mirabilis	6 (4.3)	1 (1.8)	5 (5.9)	0.40
Proteus vulgaris	0	0	0	-
Providencia stuartii	2 (1.4)	0	2 (2.4)	0.52
Pseudomonas aeruginosa	25 (18)	8 (15)	17 (20)	0.41
Raoultella onrnithinolytica	1 (0.7)	0	1 (1.2)	1.0
Serratia marcescens	4 (2.9)	2 (3.6)	2 (2.4)	0.65
Stenotrophomonas maltophilia	4 (2.9)	2(3.6)	2 (2.4)	0.65
Infection Characteristics				
Number of organisms in index culture; mean, (SD)	1.69 (0.84)	1.5 (0.72)	1.8 (0.90)	**<0.05***
Polymicrobial (>1 in index culture), n (%)	69 (49.3)	22 (40.0)	47 (55.3)	0.08*
ID Consult	138 (98.6)	54 (98.2)	84 (98.8)	1.0
Surgical consult	27 (19.6)	10 (18.2)	17 (20.5)	0.74
Surgical source control procedure (choice=Any)[a]	24 (17.1)	8 (14.5)	16 (18.8)	0.51
Antibiotic Agents				
BL/BLI (CZA/C/T/M-V)	117 (83.6)	47 (85.5)	70 (82.4)	0.63

Ceftazidime-avibactam (CZA)	76 (54.3)	29 (52.7)	47 (55.3)	0.77
Ceftolozane-tazobactam (C/T)	6 (4.3)	5 (9.1)	1 (1.2)	**0.04**
Meropenem-Vaborbactam (M-V)	45 (32.1)	19 (34.5)	26 (30.6)	0.62
Polymyxin, Colistin, or Aminoglycoside	54 (38.6)	27 (49.1)	27 (31.8)	**0.04***
Polymyxin/Colistin	22 (15.7)	12 (21.8)	10 (11.8)	0.11
Aminoglycoside (any)	37 (26.4)	18 (32.7)	19 (22.4)	0.17
Amikacin	11 (7.9)	7 (12.7)	4 (4.7)	0.11
Tigecycline	24 (17)	10 (18)	14 (17)	0.79
Carbapenem (any)	67 (48)	27 (49)	40 (47)	0.81
Ertapenem	15 (11)	5 (9.1)	10 (12)	0.62
Imipenem	0	0	0	-
Meropenem	58 (41.4)	23 (41.8)	35 (41.2)	0.94
Extended-infusion [n=54, %]	24 [44]	12 [57]	12 [36]	0.13
CRE MICs and Active Therapy				
Meropenem MICs; median (Q_1-Q_3), [n=137][b,c]	16 (4.0-16)	16 (4.0-16)	8.0 (2.0-16)	0.16
Meropenem resistant, MIC≥4 or R, [n=137][c]	104 [76]	43 [80]	61 [74]	0.41
Imipenem resistant, MIC≥4 or R, [n=57][c]	44 [79]	18 [86]	26 [74]	0.50
Ertapenem resistant, MIC≥2 or R, [n=139][c]	138 [99]	54 [100]	84 [99]	1.0
CZA resistant, MIC≥16/4 or R, [n=102][c]	7 [6.9]	5 [11]	2 [3.6]	0.24
C/T resistant, MIC≥8/4 or R, [n=0][c]	-	-	-	-
M-V resistant, MIC≥16/8 or R, [n=38][c]	1 [2.6]	1 [5.9]	0	0.45
Active therapy given[d]	131 (94)	51 (93)	80 (94)	0.74
Time to active abx, days; mean (SD) [n=131]	3.2 (4.5)	2.8 (2.0)	3.4 (5.5)	0.48
Median (IQR)	3.0 (3.0)	3.0 (3.0)	2.0 (3.0)	0.60
Range	(-4.0 to 37)	(0 to 8.0)	(-4.0 to 37)	
Within 48 hours from index culture	63 [48]	22 [43]	41 [51]	0.37
Outcomes				
**Clinical Failure**	55 (39)	55 (100)	0	-
Mortality, 30-day (index culture)	29 (20.7)	29 (52.7)	0	**<0.01**
Recurrence, 30-day (on or w/in 30 days from end of tx)	12 (8.6)	12 (21.8)	0	**<0.01**
Symptom Resolution	105 (75.0)	25 (45.5)	80 (94.1)	**<0.01**
Length of Stay, days; median (IQR)	25.5 (34)	34.0 (37)	22.0 (31)	**0.04**
Adverse Drug reaction, n (%)				
Nephrotoxicity	14 (10)	5 (9.1)	9 (11)	0.77
C. difficile	3 (2.1)	2 (3.6)	1 (1.2)	0.56
Neutropenia	2 (1.4)	1 (1.8)	1 (1.2)	1.0
None	119 (85)	47 (85.5)	72 (84.7)	0.90

CRE: Carbapenem-Resistant *Enterobacterales*, SD: Standard Deviation, IQR: Interquartile Range, BMI: Body Mass Index, COPD: Chronic Obstructive Pulmonary Disorder, PUD: Peptic Ulcer Disease, CKD: Chronic Kidney Disease, w/o: without, w/: with, AIDS: Acquired Immunodeficiency Syndrome, PWID: Persons Who Inject Drugs, Immunocompromised (APACHE-defined): Any chemo or radiation therapy within 30 days, HIV/AIDS with CD4< 200, or chronic steroids (equivalent to >40 mg prednisone), APACHE (II-Score): Acute Physiology and Chronic Health Evaluation (II score), MDR: Multi-Drug Resistant, hosp: Hospitalization, abx: Antibiotics, SOFA: Sequential Organ Failure Assessment, SIRS: Systemic Inflammatory Response Syndrome, ICU: Intensive Care Unit, IAI: Intra-abdominal Infection, SSTI: Skin and Soft Tissue Infection, UTI: Urinary Tract Infection, CLABSI: Central line-associated Bloodstream Infection, ID: Infectious Diseases, BL/BLI: Beta-lactam / Beta-lactamase Inhibitor, MIC: Minimum Inhibitory Concentration, C. difficile: *Clostridioides difficile* Infection, Neutropenia: Absolute Neutrophil Count <1500 cells/mm3 or a 50% decrease if ANC<1500 cells/mm3 at baseline

[a]Surgical source control procedures included: Intravenous catheter removal, valvular repair/replacement, invasive device removal, incision and drainage, drain placement, debridement, resection, excision, or amputation.

[b]Ordinal MICs calculated using inference of MICs where an MIC of ≤0.25=0.25, ≤0.5=0.5, <1=0.5, <4=2, >1=2, >8=16, ≥16=16, and >16=32.

[c]Available in vitro CRE MICs (Automated system, Etest, Kirby Bauer, or Broth Microdilution methods)

[d]Active therapy defined as an antibiotic given for the index infection that is non-resistant by in vitro organism MIC.

Table S2. Variance Inflation Factors, Initial – Clinical Failure

Model		Unstandardized Coefficients		Standardized Coefficients	t	Sig.	Collinearity Statistics	
		B	Std. Error	Beta			Tolerance	VIF
1	(Constant)	.387	.379		1.023	.309		
	Co-morbid conditions (choice=Peripheral vascular disease (e.g., DVT, chronic venous stasis))	-.089	.114	-.073	-.779	.438	.707	1.414
	Co-morbid conditions (choice=Connective tissue disease (e.g, osteoarthritis, rheumatoid arthritis))	-.245	.137	-.157	-1.784	.077	.813	1.230
	Co-morbid conditions (choice=Chronic dialysis (hemodialysis or peritoneal dialysis))	.353	.133	.232	2.661	.009	.827	1.209
	Patient admitted to ICU at all during admission	.061	.131	.050	.467	.642	.540	1.851
	APACHE II score: calculated	-.003	.007	-.052	-.408	.684	.385	2.596
	Pitt Bacteremia Score	.000	.024	.002	.013	.989	.420	2.380
	SOFA score	.028	.017	.214	1.642	.103	.370	2.705
	Klebsiella pneumoniae - CRE?	-.771	.531	-.736	-1.451	.149	.024	41.028
	Escherichia coli - CRE?	-.291	.277	-.215	-1.051	.295	.151	6.642
	Organism(s) (choice=Escherichia coli)	.128	.254	.101	.505	.614	.156	6.420
	Organism(s) (choice=Klebsiella pneumoniae)	.909	.524	.863	1.734	.086	.025	39.518
	Polymicrobial	-.063	.153	-.064	-.411	.682	.256	3.905
	Number of Concomitant Organisms	-.036	.091	-.062	-.398	.692	.257	3.893
	Antibiotic: Any Polymyxin, Colistin, or Aminoglycoside use	.117	.087	.116	1.336	.184	.827	1.209
	Continuous Date from earliest	1.458E-5	.000	.023	.248	.804	.705	1.418
	CRE MICs for Meropenem - Ordinal	.003	.007	.041	.427	.670	.695	1.438
	ID consult	-.232	.345	-.057	-.673	.502	.874	1.144
	source=Respiratory tract (pneumonia)	-.037	.106	-.037	-.351	.726	.568	1.760
	APACHE defined Organ System Insufficiency or Immuno-Compromised State (choice=Immuno-compromised (chemo or radiation therapy within 30 days, HIV/AIDS with CD4< 200 or on HAART therapy, or chronic steroids (>40 mg prednisone or equiv.)))	.157	.156	.087	1.006	.316	.833	1.200

a. Dependent Variable: Clinical Success = No worsening or failure to improve clinically while on drug & 30-day survival & no 30-day micro recurrence

Table S3. Variance Inflation Factors, Final – Clinical Failure

Model		Unstandardized Coefficients B	Unstandardized Coefficients Std. Error	Standardized Coefficients Beta	t	Sig.	Collinearity Statistics Tolerance	Collinearity Statistics VIF
1	(Constant)	.277	.355		.781	.436		
	Co-morbid conditions (choice=Peripheral vascular disease (e.g., DVT, chronic venous stasis))	-.099	.113	-.082	-.875	.383	.716	1.396
	Co-morbid conditions (choice=Connective tissue disease (e.g, osteoarthritis, rheumatoid arthritis))	-.216	.133	-.138	-1.624	.107	.857	1.167
	Co-morbid conditions (choice=Chronic dialysis (hemodialysis or peritoneal dialysis))	.328	.130	.216	2.524	.013	.851	1.175
	Patient admitted to ICU at all during admission	.050	.128	.041	.393	.695	.560	1.785
	SOFA score	.026	.012	.199	2.125	.036	.707	1.415
	Organism(s) (choice=Escherichia coli)	-.067	.122	-.053	-.551	.582	.667	1.500
	Organism(s) (choice=Klebsiella pneumoniae)	.177	.108	.168	1.644	.103	.593	1.687
	Number of Concomitant Organisms	-.050	.051	-.086	-.975	.331	.808	1.237
	Antibiotic: Any Polymyxin, Colistin, or Aminoglycoside use	.088	.085	.088	1.031	.305	.860	1.163
	CRE MICs for Meropenem - Ordinal	.004	.006	.055	.606	.545	.742	1.347
	ID consult	-.196	.342	-.048	-.573	.568	.883	1.132
	source=Respiratory tract (pneumonia)	-.050	.095	-.050	-.527	.599	.697	1.435
	APACHE defined Organ System Insufficiency or Immuno-Compromised State (choice=Immuno-compromised (chemo or radiation therapy within 30 days, HIV/AIDS with CD4< 200 or on HAART therapy, or chronic steroids (>40 mg prednisone or equiv.)))	.165	.155	.092	1.067	.288	.839	1.192
	Continuous Date from earliest	1.443E-5	.000	.023	.258	.797	.769	1.300

a. Dependent Variable: Clinical Success = No worsening or failure to improve clinically while on drug & 30-day survival & no 30-day micro recurrence

Table S4. Multivariate Logistic Regression Stepwise Procedure – Clinical Failure

		B	S.E.	Wald	df	Sig.	Exp(B)	95% C.I.for EXP(B)	
								Lower	Upper
Step 1[a]	Co-morbid conditions (choice=Peripheral vascular disease (e.g., DVT, chronic venous stasis))(1)	-.609	.617	.974	1	.324	.544	.162	1.823
	Co-morbid conditions (choice=Connective tissue disease (e.g, osteoarthritis, rheumatoid arthritis))(1)	-1.317	.800	2.706	1	.100	.268	.056	1.287
	Co-morbid conditions (choice=Chronic dialysis (hemodialysis or peritoneal dialysis))(1)	1.733	.766	5.123	1	.024	5.658	1.262	25.375
	Patient admitted to ICU at all during admission(1)	.224	.714	.099	1	.753	1.252	.309	5.074
	SOFA score	.133	.066	4.075	1	.044	1.142	1.004	1.300
	Organism(s) (choice=Escherichia coli)(1)	-.430	.687	.391	1	.532	.651	.169	2.503
	Organism(s) (choice=Klebsiella pneumoniae)(1)	.953	.608	2.460	1	.117	2.594	.788	8.538
	Number of Concomitant Organisms	-.287	.294	.953	1	.329	.750	.422	1.335
	Antibiotic: Any Polymyxin, Colistin, or Aminoglycoside use	.459	.440	1.087	1	.297	1.583	.668	3.751
	CRE MICs for Meropenem - Ordinal	.024	.034	.502	1	.478	1.024	.958	1.095
	ID consult(1)	-1.038	1.650	.396	1	.529	.354	.014	8.992
	source=Respiratory tract (pneumonia)(1)	-.177	.492	.129	1	.719	.838	.320	2.198
	APACHE defined Organ System Insufficiency or Immuno-Compromised State (choice=Immuno-compromised (chemo or radiation therapy within 30 days, HIV/AIDS with CD4< 200 or on HAART therapy, or chronic steroids (>40 mg prednisone or equiv.)))(1)	.884	.835	1.121	1	.290	2.421	.471	12.448
	Continuous Date from earliest	.000	.000	.146	1	.702	1.000	1.000	1.001
	Constant	-1.168	1.730	.456	1	.499	.311		
Step 2[a]	Co-morbid conditions (choice=Peripheral vascular disease (e.g., DVT, chronic venous stasis))(1)	-.684	.569	1.446	1	.229	.505	.166	1.538
	Co-morbid conditions (choice=Connective tissue disease (e.g, osteoarthritis, rheumatoid arthritis))(1)	-1.366	.785	3.027	1	.082	.255	.055	1.189
	Co-morbid conditions (choice=Chronic dialysis (hemodialysis or peritoneal dialysis))(1)	1.748	.762	5.261	1	.022	5.741	1.289	25.561
	SOFA score	.141	.061	5.241	1	.022	1.151	1.020	1.299
	Organism(s) (choice=Escherichia coli)(1)	-.413	.686	.363	1	.547	.661	.172	2.538
	Organism(s) (choice=Klebsiella pneumoniae)(1)	.921	.599	2.366	1	.124	2.511	.777	8.115
	Number of Concomitant Organisms	-.274	.291	.887	1	.346	.760	.429	1.345
	Antibiotic: Any Polymyxin, Colistin, or Aminoglycoside use	.482	.434	1.235	1	.266	1.619	.692	3.788
	CRE MICs for Meropenem - Ordinal	.026	.034	.575	1	.448	1.026	.960	1.096
	ID consult(1)	-.940	1.636	.330	1	.566	.391	.016	9.643
	source=Respiratory tract (pneumonia)(1)	-.145	.482	.091	1	.763	.865	.336	2.225
	APACHE defined Organ System Insufficiency or Immuno-Compromised State (choice=Immuno-compromised (chemo or radiation therapy within 30 days, HIV/AIDS with CD4< 200 or on HAART therapy, or chronic steroids (>40 mg prednisone or equiv.)))(1)	.823	.812	1.026	1	.311	2.278	.463	11.195
	Continuous Date from earliest	.000	.000	.161	1	.688	1.000	1.000	1.001

	Constant	-1.156	1.745	.439	1	.508	.315		
Step 3[a]	Co-morbid conditions (choice=Peripheral vascular disease (e.g., DVT, chronic venous stasis))(1)	-.653	.559	1.362	1	.243	.521	.174	1.558
	Co-morbid conditions (choice=Connective tissue disease (e.g, osteoarthritis, rheumatoid arthritis))(1)	-1.396	.781	3.198	1	.074	.247	.054	1.143
	Co-morbid conditions (choice=Chronic dialysis (hemodialysis or peritoneal dialysis))(1)	1.709	.754	5.133	1	.023	5.525	1.259	24.239
	SOFA score	.136	.060	5.224	1	.022	1.146	1.020	1.289
	Organism(s) (choice=Escherichia coli)(1)	-.365	.667	.300	1	.584	.694	.188	2.566
	Organism(s) (choice=Klebsiella pneumoniae)(1)	.947	.594	2.545	1	.111	2.578	.805	8.254
	Number of Concomitant Organisms	-.301	.279	1.168	1	.280	.740	.428	1.278
	Antibiotic: Any Polymyxin, Colistin, or Aminoglycoside use	.484	.434	1.248	1	.264	1.623	.694	3.797
	CRE MICs for Meropenem - Ordinal	.023	.033	.507	1	.476	1.024	.960	1.092
	ID consult(1)	-.913	1.615	.320	1	.572	.401	.017	9.513
	APACHE defined Organ System Insufficiency or Immuno-Compromised State (choice=Immuno-compromised (chemo or radiation therapy within 30 days, HIV/AIDS with CD4< 200 or on HAART therapy, or chronic steroids (>40 mg prednisone or equiv.)))(1)	.852	.805	1.121	1	.290	2.345	.484	11.359
	Continuous Date from earliest	.000	.000	.188	1	.665	1.000	1.000	1.001
	Constant	-1.199	1.722	.485	1	.486	.301		
Step 4[a]	Co-morbid conditions (choice=Peripheral vascular disease (e.g., DVT, chronic venous stasis))(1)	-.625	.554	1.273	1	.259	.535	.181	1.585
	Co-morbid conditions (choice=Connective tissue disease (e.g, osteoarthritis, rheumatoid arthritis))(1)	-1.393	.782	3.173	1	.075	.248	.054	1.150
	Co-morbid conditions (choice=Chronic dialysis (hemodialysis or peritoneal dialysis))(1)	1.686	.750	5.051	1	.025	5.397	1.241	23.475
	SOFA score	.141	.059	5.734	1	.017	1.151	1.026	1.292
	Organism(s) (choice=Escherichia coli)(1)	-.344	.666	.267	1	.605	.709	.192	2.614
	Organism(s) (choice=Klebsiella pneumoniae)(1)	.873	.568	2.360	1	.124	2.394	.786	7.290
	Number of Concomitant Organisms	-.289	.276	1.100	1	.294	.749	.436	1.286
	Antibiotic: Any Polymyxin, Colistin, or Aminoglycoside use	.482	.433	1.238	1	.266	1.619	.693	3.785
	CRE MICs for Meropenem - Ordinal	.023	.033	.501	1	.479	1.024	.960	1.092
	ID consult(1)	-.730	1.579	.214	1	.644	.482	.022	10.633
	APACHE defined Organ System Insufficiency or Immuno-Compromised State (choice=Immuno-compromised (chemo or radiation therapy within 30 days, HIV/AIDS with CD4< 200 or on HAART therapy, or chronic steroids (>40 mg prednisone or equiv.)))(1)	.786	.787	.999	1	.317	2.195	.470	10.260
	Constant	-1.057	1.708	.383	1	.536	.347		
Step 5[a]	Co-morbid conditions (choice=Peripheral vascular disease (e.g., DVT, chronic venous stasis))(1)	-.640	.553	1.338	1	.247	.528	.179	1.559
	Co-morbid conditions (choice=Connective tissue disease (e.g, osteoarthritis, rheumatoid arthritis))(1)	-1.401	.781	3.216	1	.073	.246	.053	1.139
	Co-morbid conditions (choice=Chronic dialysis (hemodialysis or peritoneal dialysis))(1)	1.671	.750	4.968	1	.026	5.320	1.224	23.132
	SOFA score	.141	.059	5.764	1	.016	1.152	1.026	1.292

	Organism(s) (choice=Escherichia coli)(1)	-.306	.658	.216	1	.642	.736	.203	2.677
	Organism(s) (choice=Klebsiella pneumoniae)(1)	.876	.566	2.393	1	.122	2.402	.791	7.289
	Number of Concomitant Organisms	-.296	.275	1.157	1	.282	.744	.434	1.275
	Antibiotic: Any Polymyxin, Colistin, or Aminoglycoside use	.497	.431	1.329	1	.249	1.644	.706	3.830
	CRE MICs for Meropenem - Ordinal	.022	.033	.441	1	.507	1.022	.958	1.090
	APACHE defined Organ System Insufficiency or Immuno-Compromised State (choice=Immuno-compromised (chemo or radiation therapy within 30 days, HIV/AIDS with CD4< 200 or on HAART therapy, or chronic steroids (>40 mg prednisone or equiv.)))(1)	.761	.784	.942	1	.332	2.140	.460	9.955
	Constant	-1.757	.790	4.941	1	.026	.173		
Step 6[a]	Co-morbid conditions (choice=Peripheral vascular disease (e.g., DVT, chronic venous stasis))(1)	-.645	.551	1.369	1	.242	.525	.178	1.546
	Co-morbid conditions (choice=Connective tissue disease (e.g, osteoarthritis, rheumatoid arthritis))(1)	-1.427	.778	3.368	1	.066	.240	.052	1.102
	Co-morbid conditions (choice=Chronic dialysis (hemodialysis or peritoneal dialysis))(1)	1.649	.745	4.906	1	.027	5.204	1.209	22.399
	SOFA score	.142	.059	5.833	1	.016	1.153	1.027	1.293
	Organism(s) (choice=Klebsiella pneumoniae)(1)	1.003	.499	4.031	1	.045	2.726	1.024	7.256
	Number of Concomitant Organisms	-.310	.273	1.284	1	.257	.734	.430	1.253
	Antibiotic: Any Polymyxin, Colistin, or Aminoglycoside use	.501	.431	1.349	1	.245	1.650	.709	3.842
	CRE MICs for Meropenem - Ordinal	.019	.032	.353	1	.552	1.019	.957	1.086
	APACHE defined Organ System Insufficiency or Immuno-Compromised State (choice=Immuno-compromised (chemo or radiation therapy within 30 days, HIV/AIDS with CD4< 200 or on HAART therapy, or chronic steroids (>40 mg prednisone or equiv.)))(1)	.763	.783	.950	1	.330	2.145	.462	9.953
	Constant	-1.847	.768	5.777	1	.016	.158		
Step 7[a]	Co-morbid conditions (choice=Peripheral vascular disease (e.g., DVT, chronic venous stasis))(1)	-.618	.547	1.279	1	.258	.539	.184	1.574
	Co-morbid conditions (choice=Connective tissue disease (e.g, osteoarthritis, rheumatoid arthritis))(1)	-1.323	.754	3.073	1	.080	.266	.061	1.169
	Co-morbid conditions (choice=Chronic dialysis (hemodialysis or peritoneal dialysis))(1)	1.668	.751	4.932	1	.026	5.301	1.216	23.103
	SOFA score	.149	.058	6.592	1	.010	1.160	1.036	1.300
	Organism(s) (choice=Klebsiella pneumoniae)(1)	1.079	.483	4.997	1	.025	2.941	1.142	7.574
	Number of Concomitant Organisms	-.333	.269	1.531	1	.216	.717	.423	1.215
	Antibiotic: Any Polymyxin, Colistin, or Aminoglycoside use	.508	.431	1.391	1	.238	1.662	.715	3.865
	APACHE defined Organ System Insufficiency or Immuno-Compromised State (choice=Immuno-compromised (chemo or radiation therapy within 30 days, HIV/AIDS with CD4< 200 or on HAART therapy, or chronic steroids (>40 mg prednisone or equiv.)))(1)	.696	.775	.808	1	.369	2.006	.439	9.157
	Constant	-1.712	.729	5.517	1	.019	.180		
Step 8[a]	Co-morbid conditions (choice=Peripheral vascular disease (e.g., DVT, chronic venous stasis))(1)	-.675	.547	1.526	1	.217	.509	.174	1.486
	Co-morbid conditions (choice=Connective tissue disease (e.g, osteoarthritis, rheumatoid arthritis))(1)	-1.345	.761	3.118	1	.077	.261	.059	1.159

	Co-morbid conditions (choice=Chronic dialysis (hemodialysis or peritoneal dialysis))(1)	1.804	.733	6.055	1	.014	6.071	1.443	25.534
	SOFA score	.147	.057	6.547	1	.011	1.158	1.035	1.296
	Organism(s) (choice=Klebsiella pneumoniae)(1)	1.015	.470	4.655	1	.031	2.760	1.097	6.940
	Number of Concomitant Organisms	-.360	.266	1.829	1	.176	.697	.414	1.176
	Antibiotic: Any Polymyxin, Colistin, or Aminoglycoside use	.545	.427	1.629	1	.202	1.725	.747	3.985
	Constant	-1.569	.698	5.056	1	.025	.208		
Step 9[a]	Co-morbid conditions (choice=Connective tissue disease (e.g, osteoarthritis, rheumatoid arthritis))(1)	-1.339	.746	3.222	1	.073	.262	.061	1.131
	Co-morbid conditions (choice=Chronic dialysis (hemodialysis or peritoneal dialysis))(1)	1.713	.704	5.922	1	.015	5.548	1.396	22.050
	SOFA score	.156	.057	7.596	1	.006	1.169	1.046	1.306
	Organism(s) (choice=Klebsiella pneumoniae)(1)	1.015	.472	4.619	1	.032	2.758	1.093	6.958
	Number of Concomitant Organisms	-.414	.262	2.494	1	.114	.661	.395	1.105
	Antibiotic: Any Polymyxin, Colistin, or Aminoglycoside use	.496	.422	1.380	1	.240	1.642	.718	3.755
	Constant	-1.630	.699	5.430	1	.020	.196		
Step 10[a]	Co-morbid conditions (choice=Connective tissue disease (e.g, osteoarthritis, rheumatoid arthritis))(1)	-1.240	.736	2.836	1	.092	.290	.068	1.225
	Co-morbid conditions (choice=Chronic dialysis (hemodialysis or peritoneal dialysis))(1)	1.786	.692	6.655	1	.010	5.964	1.536	23.164
	SOFA score	.157	.056	7.799	1	.005	1.170	1.048	1.307
	Organism(s) (choice=Klebsiella pneumoniae)(1)	1.130	.461	6.024	1	.014	3.097	1.256	7.637
	Number of Concomitant Organisms	-.452	.261	2.993	1	.084	.636	.381	1.062
	Constant	-1.484	.680	4.762	1	.029	.227		

a. Variable(s) entered on step 1: Co-morbid conditions (choice=Peripheral vascular disease (e.g., DVT, chronic venous stasis)), Co-morbid conditions (choice=Connective tissue disease (e.g, osteoarthritis, rheumatoid arthritis)), Co-morbid conditions (choice=Chronic dialysis (hemodialysis or peritoneal dialysis)), Patient admitted to ICU at all during admission, SOFA score, Organism(s) (choice=Escherichia coli), Organism(s) (choice=Klebsiella pneumoniae), Number of Concomitant Organisms, Antibiotic: Any Polymyxin, Colistin, or Aminoglycoside use, CRE MICs for Meropenem - Ordinal, ID consult, source=Respiratory tract (pneumonia), APACHE defined Organ System Insufficiency or Immuno-Compromised State (choice=Immuno-compromised (chemo or radiation therapy within 30 days, HIV/AIDS with CD4< 200 or on HAART therapy, or chronic steroids (>40 mg prednisone or equiv.))), Continuous Date from earliest.

Figure S1. Standard residuals by Cook's Distance – Clinical Failure

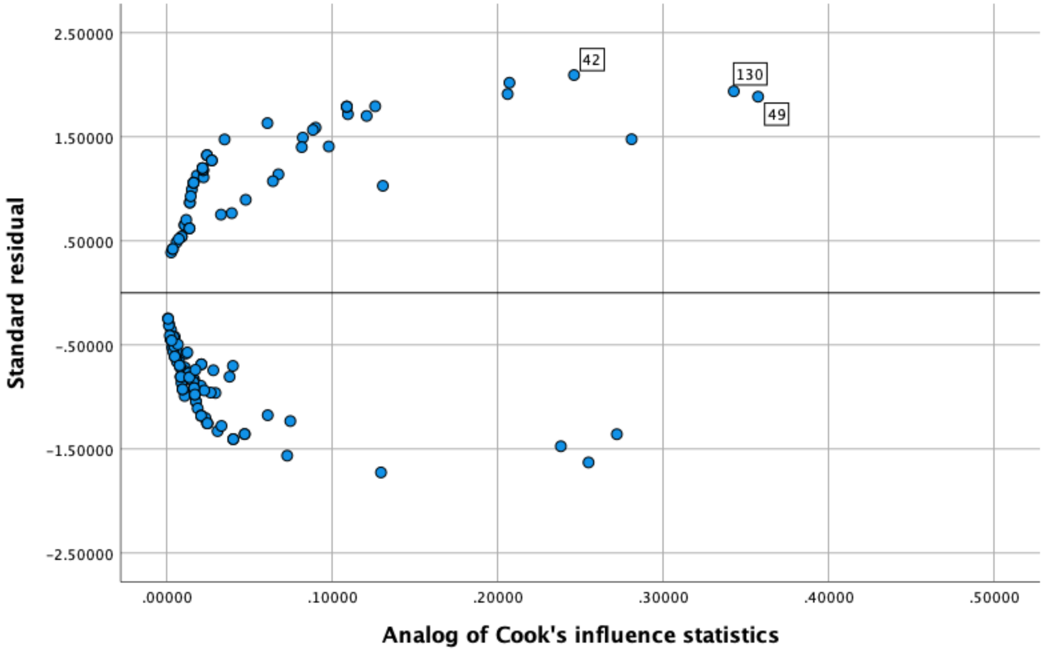

Figure S2. Standard residuals by Leverage value – Clinical Failure

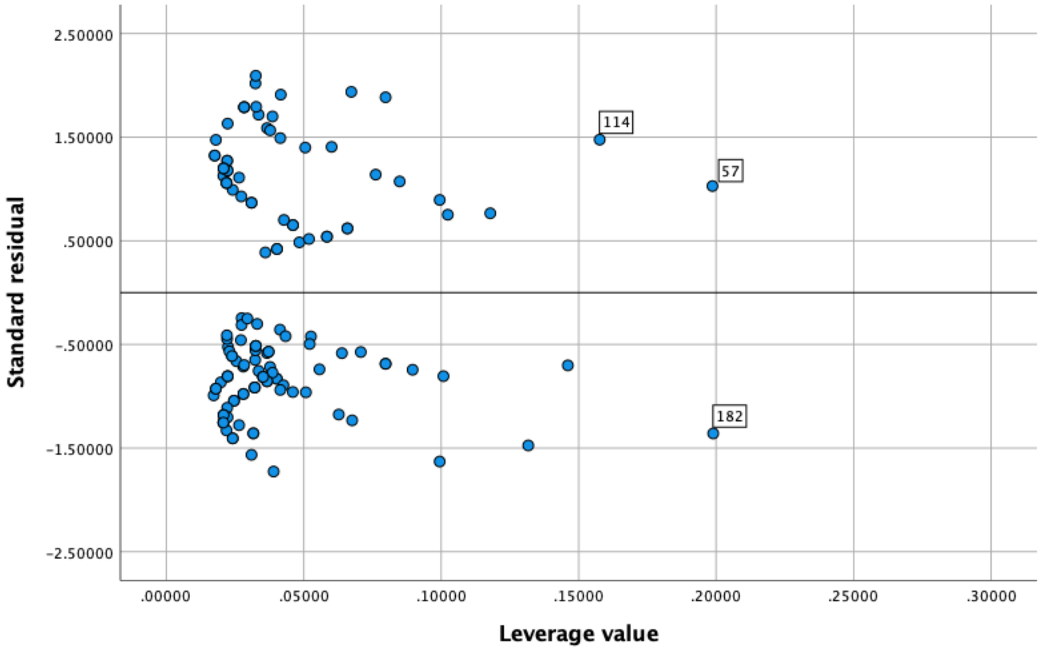

Supplement: Supplemental file 1 — Supplemental material. Download spectrum.02647-22-s0001.pdf, PDF file, 0.5 MB [file spectrum.02647-22-s0001.pdf]
